# Polycomb Directed Cell Fate Decisions in Development and Cancer

**DOI:** 10.3390/epigenomes6030028

**Published:** 2022-09-06

**Authors:** Beatriz German, Leigh Ellis

**Affiliations:** 1 Department of Medicine, Cedars-Sinai Medical Center, Los Angeles, CA 90048, USA; 2 Cedars-Sinai Samuel Oschin Comprehensive Cancer Institute, Los Angeles, CA 90048, USA; 3 Department of Biomedical Sciences, Cedars-Sinai Medical Center, Los Angeles, CA 90048, USA; 4 Center for Bioinformatics and Functional Genomics, Cedars-Sinai Medical Center, Los Angeles, CA 90048, USA

**Keywords:** polycomb group (PcG), polycomb repressive complex 2 (PRC2), the enhancer of zeste (EZH2), prostate cancer (PCa), small cell lung cancer (SCLC), Merkel cell cancer (MCC)

## Abstract

The polycomb group (PcG) proteins are a subset of transcription regulators highly conserved throughout evolution. Their principal role is to epigenetically modify chromatin landscapes and control the expression of master transcriptional programs to determine cellular identity. The two mayor PcG protein complexes that have been identified in mammals to date are Polycomb Repressive Complex 1 (PRC1) and 2 (PRC2). These protein complexes selectively repress gene expression via the induction of covalent post-translational histone modifications, promoting chromatin structure stabilization. PRC2 catalyzes the histone H3 methylation at lysine 27 (H3K27me1/2/3), inducing heterochromatin structures. This activity is controlled by the formation of a multi-subunit complex, which includes enhancer of zeste (EZH2), embryonic ectoderm development protein (EED), and suppressor of zeste 12 (SUZ12). This review will summarize the latest insights into how PRC2 in mammalian cells regulates transcription to orchestrate the temporal and tissue-specific expression of genes to determine cell identity and cell-fate decisions. We will specifically describe how PRC2 dysregulation in different cell types can promote phenotypic plasticity and/or non-mutational epigenetic reprogramming, inducing the development of highly aggressive epithelial neuroendocrine carcinomas, including prostate, small cell lung, and Merkel cell cancer. With this, EZH2 has emerged as an important actionable therapeutic target in such cancers.

## 1. Introduction

The acquisition and maintenance of cellular identity and cell fate transitions require the coordinated spatial and temporal control of gene expression programs [1,2,3]. The transcriptional diversity is principally regulated by the modulation of chromatin accessibility without changing DNA sequences, and different protein complexes behave as initiators, enhancers and/or repressors [4]. These complexes are epigenetic modifiers, which catalyze post-translational alterations in the cell by DNA methylation, histone modifications, and changes in chromatin structure and non-coding ribonucleic acids (ncRNAs) expression. Of the different epigenetic mechanisms, DNA methylation, which consists of the addition of methyl groups (CH_3_) to the C-5 position of the cytosine ring in the promoter regions of DNA, leading to the inhibition of gene expression, is the most studied [5,6]. The second epigenetic mechanism, discovered by Vincet Allfrey in the early 1960s, entails histone modifications that can promote “open” or “closed” DNA via methylation, acetylation, phosphorylation, or ubiquitination [7]. A third mechanism relates to the state of the chromatin structure, including the coiling, looping, and the general structure of DNA, which affects gene expression [8,9]. ncRNAs exert a fourth epigenetic mechanism [10,11,12] by regulating the recruitment of chromatin remodeling complexes [13], and currently, new epigenetic mechanisms, such as the methylation of RNA [14], are under investigation. Moreover, specific chromatin remodeling complexes, grouped into four families, including the SWI/SNF, CHD, ISWI and INO80 families, have the ability to displace the histone octamers from DNA or translocate them onto neighboring DNA segments [15,16,17,18], affecting gene expression.

The polycomb group (PcG) and trithorax group (trxG) are large protein multimers that are highly conserved throughout evolution, initially discovered in Drosophila melanogaster as transcriptional modulators of the Homeobox (Hox) [19,20,21]. TrxG proteins are transcriptional activators, while PcG proteins in general act as repressive regulators, and are responsible for maintaining embryonic chromatin landscapes in a poised transcriptional state throughout development [22,23,24,25]. For example, early embryos present an initial widespread loss of DNA methylation that is reacquired during implantation [26]. Moreover, in embryonic stem cells (ESCs), specific lineage and differentiation genes, including Brachyury (a T-box transcription factor required for the mesoderm formation) and *Otx2* (gene responsible of the mediation of the early differentiation and formation of the neural ectoderm), are repressed during pluripotency by polycomb repressor complex 2 (PRC2), suggesting that epigenetic marks have fundamental roles in developmental decisions [27]. More recently, it has been shown that these protein complexes play a key role in ensuring dynamic gene regulation in adult stem cell states. Therefore, PcG proteins are involved in a wide range of biological processes such as differentiation, stem cell plasticity and cell cycle progression [22]. In addition, the dysfunction of epigenetic regulation within a cancer cell has proven to be a critical mediator of tumoral initiation, progression, cell plasticity and therapy resistance, including tumor immune escape [28]. For example, it has been reported that the epigenome of tumoral cells in children with rhabdoid brain tumors [29] and in adult patients with chronic lymphocytic leukemia [30] affects the phenotype of the disease, and the therapeutical response in patients.

In this review, we first describe the molecular mechanisms underlying the function and recruitment of PcG proteins in gene regulation during normal development, with a special focus on PRC2. We then summarize the involvement of the PRC2 complex in cell differentiation during development and cancer, with a special focus on the description of the latest discovered mechanisms linking polycomb to cell fate determination, metabolism, and immune response in prostate cancer (PCa), small cell lung cancer (SCLC) and Merkel cell carcinoma (MCC). Moreover, due to the low efficiency of epigenetic modulators as monotherapy, we summarize the results of clinical trials using epigenetic treatments combined with other therapeutical options in cancer. Finally, we provide a deeper insight into the clinical potential of the subsequent inhibition of epigenetic mechanisms in combination with checkpoint inhibitors (CPI) to increase tumor immunity and the success of therapies against tumors.

## 2. PcG Complexes

**Figure 1:**
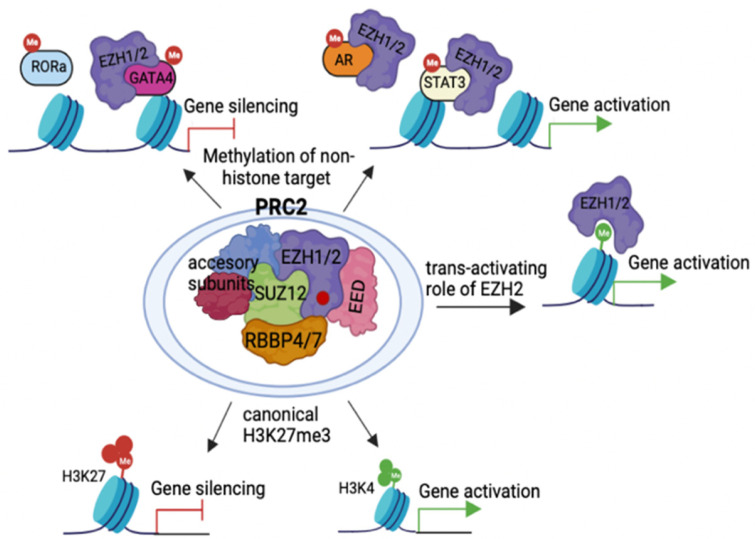
**EZH2 transcriptional regulatory activity.** PRC2 catalyzes the methylation of Histone 3 on lysine 27 or lysine 4 through its enzymatic subunit EZH1 or EZH2. H3K27me3 correlates with gene silencing and H3K4me3 correlates with gene activation. In addition, EZH2 is able to methylate several non-histone protein substrates, including GATA4, RORα, STAT3 and AR, contributing to either transcriptional silencing or activation. EZH2 also plays a PRC2-independent role in transcriptional activation. Figure generated in BioRender.com.

The two main PcG complexes identified in mammals are polycomb repressive complex 1 (PRC1), principally composed of RING1A/B and B lymphoma Mo-MLV insertion region 1 (BMI1), and 2 (PRC2) [31,32]. These two complexes act non-redundantly in the same target genes, with the objective of maintaining gene repression via post-translational modifications of histone proteins [33]. PRC1 has E3 ligase activity, which monoubiquitinates the histone H2A at lysine 119 (H2Aub1) [34], while PRC2 has a methyltransferase activity that mono-, di- or tri-methylates the lysine 27 of the histone H3 (H3K27me1/2/3), classified as transcriptionally repressive marks [35,36]. The three H3K27 methylation forms are mutually exclusive, and form spatially defined genomic domains. Whereas H3K27me1 is accumulated in the intragenic regions of actively transcribed genes, H3K27me2 forms are presented in intergenic and intragenic domains, suggesting their role in the prevention of inappropriate promoter activity. An approximate 70% of total histone H3K27 is demethylated, and 4% occurs in the trimethylated form, promoting its protective functions against changes mediated by Histone Acetyl Transferase [37]. Although imaging studies showed that PRC-repressed chromatin has a compact conformation [38,39,40], this inactive state can be reverted by the recruitment of remodeling complexes and histone modifying enzymes, including acetylation at the lysine 27 residue, resulting in a permissive active transcription, making H3K27’s post-translational modification status an important regulator of the cellular transcriptome critical for lineage commitment and the maintenance of cell identity. In addition to H3K27 acetylation, H3K4 trimethylation (H3K4me3) and H3K4 mono-methylation (H3K4me1) are other transcriptional activation marks [41,42,43,44,45] (Figure 1). Moreover, genome-wide analyses of RNA polymerase II (RNAPII) and polycomb occupancy in mouse embryonic stem cells (mESCs) have shown that PRC-bound genes contain genes actively transcribed by RNAPII, suggesting that PRC promotes chromatin bivalency [46,47]. 

The core PRC2 complex, which is conserved from flies to mammals and plants, is composed by the catalytic subunit enhancer zeste homolog 1/2 (EZH1/EZH2), the scaffolding subunits embryonic ectoderm development (EED) (which contains a WD40 repeat domain that recognizes trimethylated peptides), the suppressor of zeste 12 (SUZ12) (which is a Zinc finger-containing protein), and the retinoblastoma-associated protein (RBBP) 4/7 (also known as RbAp46/48) (review in [48]). The PRCs components in different species, including mammals, drosophila and Arabidopsis, are summarized in Table 1.

### 2.1. Action Modes of PRC2 Molecules

Although there are four core subunits, only SUZ12, EED and EZH1/2, are required for the basic function of the complex [51,54]. Both EZH proteins, which are mutually exclusive within PRC2, have five domains, and the carboxy-terminal suppressor of variegation 39 enhancer of zeste and Trithorax domain (SET domain) is responsible for the histone methyltransferase activity. Mechanistically, the SET domain transfers a methyl group from the co-factor S-adenosyl methionine (SAM), which is converted to S-adenosyl homocysteine (SAH) [55]. Despite the fact that, between EZH1 and 2, the differences in the amino acid sequence of the stimulatory responsive domain are minor, they are responsible for the higher activity of EZH2 compared with EZH1 under the same conditions [56]. While EZH2 is highly expressed in proliferating cells, EZH1 shows abundant expression in nondividing cells, which is linked to differentiated post-mitotic conditions [56]. Moreover, EZH1 phosphorylation leads to degradation, whereas EZH2 phosphorylation typically promotes reduced function [57]. In addition, it has been reported that EZH2 phosphorylation by cyclin-dependent kinase 1 (CDK1) and CDK2 at threonine 350 (T350) plays an important role in the recruitment of EZH2 to the promoters of its target genes [58], such as the ncRNAs HOTAIR and XIST [59,60]. Indeed, it has been reported that EZH2 phosphorylation by CDK2 at threonine 416 (T416) correlates with poor prognosis in triple-negative breast cancer (TNBC) and promotes cell migration and invasion, as well as tumor formation [61]. In general, methyltransferase activity is mediated by the interplay between EED and EZH2 via Van der Waals interactions and hydrogen bonds, which induce a rotational change in the PRC2 configuration [62], while the SUZ12 subunit is involved in the stabilization of the complex [63]. Finally, RBBP4/7 plays a role in regulating and promoting the stabilization of PCR2 chromatin interactions by binding to the unmethylated histone H3K4 tail impaired when H3K4me3 is present [64]. Moreover, it has been shown that the loss of and mutations in EED, EZH2, and SUZ12 promote defects during gastrulation in development, and are implicated in the development of different cancers [65]. 

In addition, posttranscriptional modifications, or differential splicing, which are less explored, have also been related with histone modification functions. Besides H3K27me3 regulation, it has been reported that EZH2 enacts a PRC-2-independent activity by methylating non-histone targets or directly interacting with non-histone proteins to activate downstream genes (Figure 1). For example, EZH2 can directly methylate and repress the transcription of the transcription factor GATA4 at Lys299, attenuating p300-mediated GATA4 acetylation [32,66] (Figure 1). Moreover, EZH2 can also modulate RAR-related orphan receptor alpha (RORα) at Lys38, promoting targeted gene silencing [67], and Gunawan et al. showed that EZH2 methylates talin, a cell migration regulatory molecule, disrupting the binding between talin and F-actin, which regulates the turnover of adhesion structures [68]. In glioblastoma, EZH2 can be phosphorylated at Ser21 by protein kinase B (AKT), leading to the methylation of STAT3 and the consequent activation of this signaling pathway [69,70,71], and in castration-resistant PCa (CRPC), phosphorylated EZH2 activates the androgen receptor (AR) via a non-catalytic mechanism directly occupying the AR promoter and activating downstream genes [71] (Figure 1). The role of EZH2 in PCa progression will be discussed in detail in the following section.

**Figure 2:**
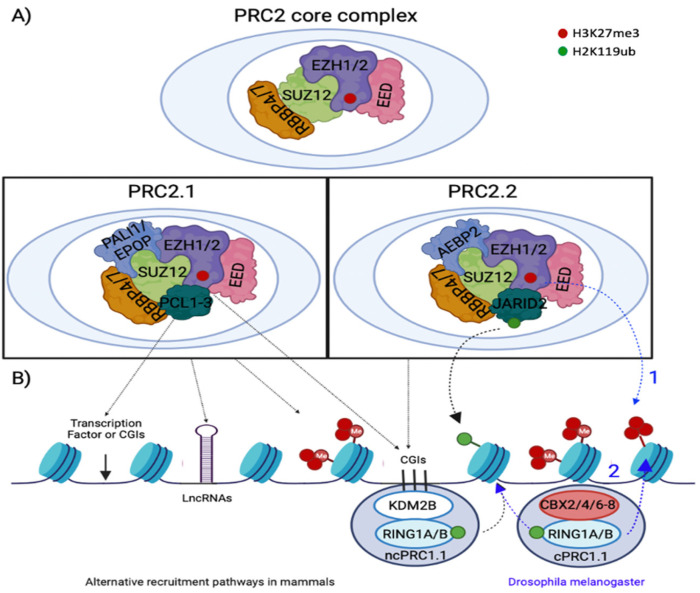
**PRC2 composition and normal mechanism of recruitment.** Scheme of PRC2 subunits in mammals and drosophila melanogaster, including the core and accessory subunits of PRC2 complex in mammals. (**A**) Subunits of PRC2.1 and PRC 2.2 protein complex in mammals. The core PRC2.1 contains PCL1/2/3 and the PALI1/2 or EPOP subunits. The binding between the core PRC2, JARID2 and AEBP2 constitutes PRC2.2. (**B**) The original pathway of PcG recruitment, first discovered in Drosophila melanogaster, has two sequential steps (represented in blue). First, PRC2 is recruited to the chromatin areas with Polycomb Response Elements (PREs) and deposits the repressive H3K27me3 mark via EZH1/2. This mark is recognized by canonical PRC1 (cPRC1) and RING1A/B deposits the ubiquitination on H2AK119. In addition, in mammals (represented in black), PRC2 recruitment occurs at CGIs, via transcription factors, lncRNAs, or an alternative recruitment pathway, in which non-canonical PRC1 (ncPRC1) complexes are recruited in a KDM2B-dependent manner, which deposits the H2AK119 ubiquitination mark recognized by the JARID2 and PCL1/2/3 subunits of PRC2.2 and PRC2.1, respectively. Figure modified from [72] and generated in BioRender.com.

PRC2 is subdivided into two types, depending on the interactions with the differentially expressed accessory subunits, PRC2.1 and PRC2.2, which influence the function of the complex and the chromatin recruitment [72] (Figure 2A). The PRC2.1 complex comprises three paralogous polycomb-like (PCL) proteins PCL1/2/3, also called PHF1, MTF2 and PHF19, as well as Elongin B/C and PRC2-associated protein (EPOP), or PRC2-associated LCOR isoform 1/2 (PALI1/2). In addition, PRC2.2 includes Jumonji, AT/rich interaction domain containing 2 (JARID2), and the Adipocyte Enhancer-Binding Protein 2 (AEBP2) (Figure 2A and Table 1) (reviewed in [73]). Several studies have suggested that while PRC2.1 is involved in the de novo recruitment of PRC2 to CpG islands that lack H3K27m3 [74,75], PRC2.2 is involved in the methyltransferase activity through recruitment to chromatin that has PcG-dependent modifications, including H3K119ub [76] or H3K27m3 [77].

### 2.2. PRC2 Recruitment

The mechanisms of the recruitment of PcG proteins to their target genes, especially in mammals, remain incompletely understood. An important feature of PRC1 and PRC2 recruitment is the absence of sequence-specific DNA-binding domains. Therefore, PcG-mediated gene regulation depends on the accessory subunits and transcription factors that direct their recruitment to specific chromatin domains. In addition, once H3K27 is trimethylated, the histone state needs to be maintained to avoid improper transcription, and this process is regulated by a positive feedback loop post initial PRC2 methyltransferase activation. Initially in Drosophila melanogaster, it was discovered that the original pathway of PRC2 recruitment has two sequential steps, and both PRC complexes have an interdependent association [78,79,80]. First, PRC2 is recruited to the chromatin by cis motifs, denominated as Polycomb Response Elements (PREs), to deposit the repressive H3K27me3 mark via EZH1/2. This mark is recognized by the canonical PRC1 through its chromodomain-containing CBX homolog, and RING1A/B mediates the ubiquitination of lysine 119 at histone 2A (H2AK119ub1) (Figure 2B). However, in mammals, although PRC2 can be recruited by PREs, only two have been identified as functional, and genome-wide analyses have indicated a strong overlap between PcG proteins and CpG islands (CGI), areas of DNA that have a high abundance of cytosines (C) and guanines (G) (reviewed in [81]). This recruitment process was denominated the “scanning model”, and it was described that the extended homologous (EH) region of PCL proteins binds with unmethylated CpG-containing DNA sequences, promoting PRC2 binding and stabilization [80,82,83,84]. In addition, JARID2 and AEBP2 regulate the recognition of H2AK119ub and H3K27me3 deposition at specific PcG target regions [85]. More recent publications have shown the existence of an alternative recruitment pathway, in which Lysine Demethylase 2B (KDM2B) proteins recognize CpG islands and recruit non canonical PRC1 (ncPRC1) complexes, which deposit the H2AK119 ubiquitination mark [86] (Figure 2B). Additionally, long non-coding RNAs (lncRNAs), including 122 lncRNA and H19 in PCa [87], as well as specific transcription factors are implicated in PRC2 recruiting mechanisms [31,88]. Nevertheless, how the multiple PcG complexes work together and cooperate in the chromatin context needs to be further defined. 

## 3. PcG Protein Functions and Cell Fate Determination

Within an organism, during development, it is fundamental that gene expression is regulated in a spatiotemporal fashion, and that only the specific programs required for cell specialization or organogenesis are active to assure homeostatic functions. Alterations in these procedures to escape from the state of terminal differentiation promote cellular plasticity [89]. Much of the knowledge about the function of PRC complexes is based on studies performed in ESCs, and in this context, PRC integrity is important to ensuring the maintenance of cell-specific transcriptional programs. For example, the early embryonic lethality of PRC knockout mice [90,91,92,93], as well as failure in the establishment of in vitro cultures from ESCs lacking PRC core subunits [63,91,94,95,96], has demonstrated the relevance of these complexes in development. Moreover, despite the fact that both EZH1/2 are expressed in mouse ESCs, the loss of EZH1 is related to embryogenesis and mouse viability [97], but it fails to compensate for the EZH2 loss of function that results in early embryonic lethality during gastrulation [90,91,98,99,100]. In mouse ESCs, the PRC target repressed genes display opposite chromatin methylation patters, denominated “bivalent domains”, including Lys27 and Lys4 methylated regions. Upon differentiation, ESC’s developmental genes are selectively enriched for either Lys27 or Lys4 methylation [42]. These modified regions provide a robust epigenetic memory to maintain lineage-specific expression or repression profiles, which are different between pluripotent ESCs, embryonic carcinoma cells, hematopoietic stem cells (HSCs), and their differentiated progeny [41]. For example, in PCa, the transcriptional embryonic prostatic program is activated; therefore, tumoral cells regain a bivalent chromatin state, allowing cells to switch identity or increase stemness [101]. The loss of PRC function, caused by abnormal expression, translocation, misrecruitment or genetic mutations that preferentially target PRC2 subunits affecting the activity of the complex, causes global changes in gene expression, promoting the initiation of alternative pathways. PRC2 mutations are frequent in hematological cancers. 

**Figure 3:**
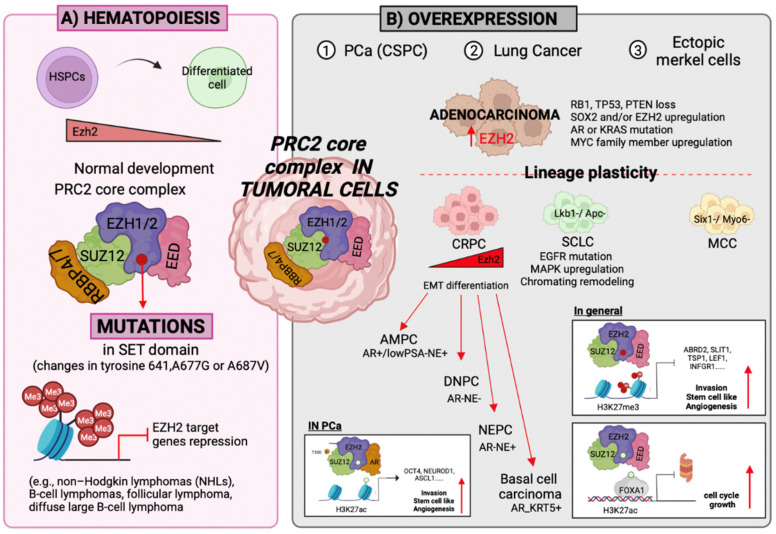
**PRC2 alterations in cancer development.** (**A**) Dynamic of the PRC2 complex during hematopoietic stem/progenitor cells’ (HSPCs) differentiation and cancer development. Throughout the normal differentiation process of hematopoietic stem/progenitor cells (HSPCs), EZH2 expression is decreased from HSPCs to differentiated cells. Mutations in PRC2 subunits, including EZH2 (e.g., mutations in the SET domain produced by changes in tyrosine 641A, A677G or A687V), promote H3K27-trimethylation, leading to a strong repression of EZH2 target genes and the development of non-Hodgkin lymphomas (NHLs), B-cell lymphomas, follicular lymphoma, and diffuse large B-cell lymphoma. (**B**) In contrast, it has been reported that EZH2 is upregulated in neuroendocrine cancers, including prostate cancer (PCa), lung adenocarcinoma and Merkel cell carcinoma (MCC). In PCa, the EZH2 expression level increases during tumoral development from castration-sensitive PCa (CSPC), which is characterized by the expression of androgen receptor (AR) and prostate-specific antigen (PSA), to therapeutical resistant tumors (CRPC), subclassified into amphicrine PCa (AMPC), double-negative PCa (DNPC), neuroendocrine PCa (NEPC) and basal cell carcinoma. EZH2 expression promotes tumoral transition via multiple mechanisms, including its canonical transcription repression activity via PRC2 to promote tumor cell invasion, stem cell features, and angiogenesis. Moreover, in PCa, EZH2 interacts with the AR promoter, increasing the transcriptional activity of AR at the early tumoral stage. During tumoral evolution, EZH2 can act as a co-repressor, with N-MYC suppressing canonical AR signaling. In addition, it has been shown that pEZH2-T350 interacts with AR, promoting stem cell plasticity, angiogenesis, and neuronal processes. Finally, EZH2 mediates the maintenance of PCa independently of AR signaling. Lung adenocarcinoma can evolve to small cell lung cancer (SCLC), undergoing a squamous transformation following epigenomic reprogramming due to the loss of *Lkb1-*, *Apc-* and PRC2-related H3K27me3 repressive chromatin marks. Moreover, EZH2 is overexpressed in 54% of MCC and is associated with a poor prognosis, and its inhibition reduces the tumoral growth in xenografts, derepressing SIX1 and MYO6 expression. Figure generated in BioRender.com.

In normal development, EZH2 interaction with BCL6 is necessary for immunoglobulin rearrangement [102] and germinal center formation by silencing *p21/p27* and *Blimp1* loci [103,104,105]. While *EZH2* deletion suppresses the germinal center formation, in B-cell lymphomas, EZH2 gain-of-function in the SET domain, including changes in tyrosine 641, A677G or A687V, promotes alterations in the substrate-binding pocket, enhancing H3K27me3 and decreasing H3K27me2 [105,106,107,108,109]. These mutations mechanistically promote the repression of EZH2 target genes and lead to the disruption of cell fate control, allowing lymphoma cells to stop the normal B-cell maturation process and stay in a highly proliferative state, leading to germinal center hyperplasia [103,104,105,110] (Figure 3A). In addition, the heterozygous substitution of the K27 residue of H3.3 into a non-methylatable methionine has been identified as a driver mutation in pediatric glioblastomas [111]. However, in most of the solid tumors, including endometrial, PCa, lung, breast cancer and melanoma [112,113,114,115], EZH2 is most often overexpressed, leading to a variant PRC that may alter the control of the target gene’s expression level [116] (e.g., high levels of H3K27me3, associated with the overexpression of EZH2, correlate with poor prognosis in PCa and lung cancer [117,118]). In addition, accumulation evidence indicates that switch/sucrose non-fermentable (SWI/SNF) remodeling complexes repress the epigenetic silencing by PcG proteins, and mutations in this complex lead to the indirect reprograming of PRC2 in tumors [119,120,121,122]. For instance, in mesenchymal TNBC and malignant rhabdoid tumors, which arise in the brain, kidney, and other soft tissues, the loss of SNF5 (INI1) results in the altered genomic deposition of the PRC2 complex in H3K27me3 residues, leading to the repression of lineage-specific target genes [122]. In mesenchymal TNBC, EZH2 expression is not affected, suggesting that PRC2 activation derives from indirect regulation [122]. Moreover, the *SMARCE1* gene, which encodes a core subunit of SWI/SNF, is highly expressed, and has been associated with poor prognosis in patients with neuroblastoma. Mechanistically, SMARCE1 directly interacts with MYCN, which is necessary for the MYCN-mediated transcriptional activation of downstream target genes, including *PLK1*, *ODC1*, and *E2F2* [123].

As described in the latest Hallmarks of Cancer review, phenotypic plasticity is a critical component of cancer pathogenesis, and the loss of cellular differentiation leads to the acquisition of a set of functional capabilities and a stem cell-like phenotype characterized by uncontrolled growth, intratumoral heterogeneity and metastasis [124]. Therefore, tumor evolution is driven by the acquisition of genetic aberrations (e.g., *Rb1* or *Trp53*) accompanied by significant chromatin remodeling [34,125,126]. These epigenetic changes lead to cellular switching to an alternate transdifferentiation program and cellular dedifferentiation to a progenitor-like cell state, and neoplastic cells arising from a progenitor cell will not continue with the differentiation program [111,124,127,128,129,130]. Therefore, epigenetic therapies are a promising therapeutical strategy in multiple cancer types [34,127,131,132]. Neuroendocrine carcinomas, which can arise from almost all epithelial organs, are characterized by phenotypic plasticity and acquired therapeutic resistance, and include PCa, SCLC and MCC [133,134] (Figure 3B). PRC2 dysregulation in cancer has been studied since Varambally et al. demonstrated the association between EZH2 overexpression and the poor prognosis of PCa patients in advanced disease [117]. In the context of KRAS-driven lung adenocarcinoma, tumoral cells lose their initial stable alveolar-type2-like state and undergo a squamous transformation following epigenomic reprogramming programs due to the loss of *Lkb1*, *Apc* and PRC2-related H3K27me3 repressive chromatin marks [135,136], which promotes the upregulation of *Ngfr*, *Sox2*, Δ*Np63*, and *Krt5/6* [137,138]. EZH2 is upregulated in squamous tumors compared to adenocarcinomas, and the overall reduction in PRC2 activity is related with EED downregulation. Moreover, EZH2 is overexpressed in 54% of MCCs and is associated with a poor prognosis [139], and its inhibition reduces the tumoral growth in xenografts, derepressing SIX1 and MYO6 expression—transcription factors related to cell viability [140]. In this context of cell identity maintenance and cell-fate determination procedures, three main mechanisms have been identified, including self-renewal, quiescence, and differentiation.

### 3.1. Self-Renewal

PRC complexes maintain cell cycle regulators, including p16Ink4a, in a repressive state to prevent cell cycle exit, cell cycle arrest, and p53-mediated cell death [129,130]. PCR2 plays a major role in the self-renewal of HSCs, given that PRC2-EZH1/2 complexes promote cell survival allowing continuous bone marrow transplantation in mice [141]. While EZH2 and SUZ12 are highly expressed in both fetal and adult bone marrow, EZH1 is preferentially expressed in adult HSCs [142,143,144]. Consistent with EZH1/2’s patterns of expression, while the inactivation of EZH2 in HSC at the adult stage leads to defects in B cell maturation, the loss of EZH1 causes impaired B cell development [102,144,145], suggesting that EZH1 can compensate for EZH2 loss and maintain self-renewal capacity only at the HSC stage [102,144]. However, PcG complexes are not required for the self-renewal of ESCs, and the maintenance of pluripotency network is sufficient to avoid the exit of the cell cycle. Moreover, it has been reported that PcG protein complexes play an important role in the physiological expansion or regeneration of multiple tissues. For example, EZH2 expression is required in pancreatic islets to regulate histone methylation, leading to the repression of the Ink4a/Arf locus, allowing physiological islet β-cell expansion in neonatal mice, and β-cell regeneration after conditional chemical ablation of β-cells in adults [146]. In mouse skin, it has been reported that EZH1/2 have a redundant role, while they are indispensable for hair follicle morphogenesis and degeneration due to defective proliferation, increased apoptosis, and fully activated Ink4a/Inkb/Arf locus; the epidermis hyperproliferates and survives engraftment [97]. In adult skeletal muscle it has been reported that EZH2 is expressed in both Pax7^+^/Myf5^−^ stem cells and Pax7^+^/Myf5^+^ committed myogenic precursors, and is required to maintain the cellular proliferation of satellite cells responsible for muscle regeneration and the maintainance of muscle mass [147]. In the intestinal epithelium, RPC2 is required to sustain progenitor cell proliferation in the crypt bottom cells, mediated by the derepression of Cdkn2a expression, and the correct balance between secretory and absorptive lineage differentiation programs by the direct regulation of the expression of ATOH1 and GFI1, direct transcriptional controllers of secretory lineage cells and goblet cell specification [148].

In B and T-cell lymphomas, it has been shown that BMI1 (a subunit of PRC1, which promotes self-renewal in skeletal muscle [149]) acts as a proto-oncogene inhibiting Myc-induced apoptosis through repression of the cyclin-dependent kinase inhibitor 2A (*Cdkn2a*), the gene responsible for the expression of INK4 and ARF proteins, which restrict cellular proliferation [150]. Therefore, INK4 inhibition affects retinoblastoma (RB) pathway activation and ARF cannot induce p53 by inhibiting MDM2 activity [151,152]. More recently, Shields et al. demonstrated that the depletion of *BMI1*, which is implicated in the transformation of normal human myoblasts into alveolar rhabdomyosarcoma (ARMS), delays tumor growth via activation of the Hippo pathway by LATS1/2 phosphorylation, leading to reduction in YAP levels and YAP/TAZ target genes [153,154]. Moreover, EZH2 showed an oncogenic role in promoting tumoral growth and cell invasion in breast cancer by the inhibition of E2F1-dependent apoptosis pathway by the epigenetic modulation of *BIM* expression [93]. In addition, it has been reported that in breast cancer, TGF-β enhances cancer stem cell plasticity by reducing the presence of H3K27me3 at the *ZEB1* (zinc finger E box-binding homeobox 1) promoter site [155]. In pancreatic cancer, the overexpression of EZH2 represses the cyclin-dependent kinase (CDK) inhibitor *p16Ink4a* and controls the proliferative potential of pancreas/duodenum homeobox protein 1 (PDX1), positive progenitor cells that accumulate transiently in metaplastic lesions [156]. In PCa and lung cancer, we have seen the presence of a small population of cancer stem cells with stem cell-like characteristics, which overexpress EZH2 and selectively downregulate miR-34a and *let-7b* expression [157,158]. The downregulation of miR-34a, which directly targets stem cell-related genes such as *CD44* and *NOTCH*, is crucial to maintaining cancer stem cell phenotypes [159]. Initially, in PCa, EZH2 methylates AR to promote AR transcriptional activity by PRC2-methylation dependently or by PRC2-independent mechanisms, directly occupying the *AR* promoter [71,160]. The presence of cancer stem cells became relevant due to the approximate 20% of advanced therapy-resistant PCa adenocarcinomas, and the 5% of EGFR mutant lung adenocarcinoma trans-differentiating to a neuroendocrine (NE) phenotype [161,162]. Prostate and EGFR-mutant lung adenocarcinoma initially respond to the first generation of antiandrogens (ADT), which inhibit the expression of AR signaling pathways, one of the principal drivers of PCa progression [160] and EGFR inhibitors, respectively. However, these therapies are suppressive rather than curative, and tumors acquire resistance. As represented in Figure 3B, castration-sensitive prostate cancer (CSPC) after the first line of treatment relapse, and become castration-resistant PCa (CRPC) [70]. In summary, the epigenetic modifications in tumoral cells related with self-renewal promote the acquisition of a persistent proliferative ability, resistance to cell death mechanisms and the bypass of cellular senescence programs, and tumoral cells have a higher migratory/invasive potential.

### 3.2. Differentiation

PRC has been demonstrated to be critical for the repression of developmental genes to prevent cell differentiation. In fact, it has been reported that the loss of PRC2 in ESCs is enough to induce spontaneous differentiation through the meso-endoderm germ layers without neural ectoderm [163]. Genome-wide analyses showed that PRC proteins are enriched in the promoter areas of lineage-specific transcription factors in ESCs [164,165], accompanied by H3K27me3 and H3K4me3 enrichment, suggesting as mentioned before that PRC2 plays an important role in controlling multiple transcriptional states [69]. Moreover, the loss of PRC2 in naive ESCs results in an induction of genes that regulate development, suggesting that pluripotency genes dominate over lineage-determining genes in this context [95,98]. In adult stem cells, PRC complexes maintain their HSC identity, suppressing lineage-specific gene expression [166,167], but the heterozygous depletion of EZH2, SUZ12, or EED increases hematopoietic stem progenitor cell (HSPC) activity [143,168]. Nonetheless, *SUZ12* and *EED* hematopoiesis-specific KO models induce exhaustion in HSC at the fetal or adult stage, rather than hyperproliferation, suggesting that the effect of PRC2 on HSC activity is dosage-dependent [142,169,170]. In addition, SUZ12 is essential for T and B cell maturation, but dispensable for proper myelopoiesis [169]. In leukemias, PRC complexes interact with fusion proteins and play important roles in cancer development [171,172]. Moreover, it has been shown that while JARID2 KO models affect HSPC proliferation, the depletion of PHF1, MTF2, or PHF19 does not affect HSPC [48,173,174,175]. However, MTF2 expression is necessary for proper erythrocyte maturation, including the regulation of the Wnt signaling pathway and its downstream targets *Gata2*, *Fli1*, *Myb*, and *Stat5b* [176]. Thus, these studies show that PRC2 is necessary for the long-term maintenance and maturation of hematopoiesis. In addition, in human epidermal homeostasis, it has been reported that EZH2 expression is elevated in differentiated suprabasal layers and in primary human keratinocytes (NHEKs) in vitro. While EZH2 and EED inhibition promotes NHEKs differentiation, EZH1/2 inhibition reduced cell proliferation and promoted apoptosis by upregulation of terminal differentiation genes, such as *Filaggrin*. Moreover, it has been shown that EZH2 expression is downregulated in aged keratinocytes, accompanied by the upregulation of senescence-associated genes, suggesting EZH2 involvement in epidermal aging [177]. However, despite the role of PRC2 in hair follicle self-renewal, as previously discussed, genome-wide transcriptional studies in adult hair follicular stem cells have demonstrated that PRC2 does not alter the homeostatic hair cycle, and in quiescent cells does not promote fate switch. Therefore, PRC2 and H3K27me3 play a non-instructive role in this context, and there may be redundant or alternate mechanisms acting to preserve hair follicle stem cell functions and fate maintenance [178].

In PCa, CRPC tumors can continue the transition to more aggressive disease states via lineage plasticity, and adopt a phenotype no longer reliant on AR expression and signaling. It has been reported that the EZH2-T350 phosphorylation and consequent AR interaction plays an important role in the transition and differentiation from CRPC to more aggressive PCa phenotypes [176]. Therefore, these tumors become enzalutamide resistant, a second-generation ADT, and display neuroendocrine (NE) features [179]. They are associated with high levels of chromogranin A, enolase and synaptophysin in serum, accompanied by a reduction in prostate-specific antigens (PSAs), a stem or basal cell-like phenotype, altered kinase signaling, and/or characteristic epigenetic alterations. In this scenario, EZH2 plays a key role in tumoral development, and the different disease states can be classified as amphicrine PCa (AMPC), which expresses AR but has a decreased canonical AR signaling pathway and more NE features, double-negative PCa (DNPC) that does not express AR or neuronal markers, terminal neuroendocrine PCa (NEPC) that expresses neural markers, and squamous cell carcinoma that turns on the basal cell markers, such as Krt5 or p63 [180,181] (Figure 3). NEPC is associated with aggressive clinical features and poor overall survival [182]. Clinical and preclinical studies have demonstrated that NEPC frequently involves the amplification of *MYCN* (encoding N-Myc, an oncogene associated with poor prognosis in neuroblastoma) and *AURKA* (Aurora-A) [183,184,185]. EZH2 interacts with the N-Myc/AR complex; therefore, N-Myc redirects EZH2 activity to N-Myc target genes, resulting in transcriptional repression [183,186,187], leading to AR signaling suppression and establishing other tumoral pro-survival signaling pathways. Moreover, it has been reported that, similar to CRPC, SCLC presents combinatorial loss-of-function mutations in key tumor suppressor genes, including *PTEN*, *TP53*, and *RB1* [188], suggesting cooperation between *RB1* and *TP53* in treatment resistance. The gene expression analysis of triple KO mouse tumors and human PCa patient samples with these mutations revealed altered expressions of E2F target genes and neuroendocrine lineage genes, together with increased expressions of stemness and epigenetic reprogramming-related genes, such as *SOX2* and *EZH2* [189,190]. In addition, the epigenomic profiling of histone modifications (H3K27ac, H3K4me3 and H3K27me3) in human NEPC and selected LuCaP PCa patient-derived xenografts showed a significant reprogramming of the master transcriptional regulator Forkhead box 1 (FOXA1) and an increase in H3K27ac at the *FOXA1* binding site, related to the promotion of neuroendocrine lineage-defining genes [191]. Recently, FOXA1 has emerged as an enzalutamide-induced reprograming factor in high-risk PCa patients. Linder et al. reported that AR inhibitory therapy induces the cistromic repositioning of FOXA1-promoting ARNTL expression level, a classical circadian rhythm regulator, which compensates for AR inhibition and induces cellular proliferation signals [179]. In addition, ATAC-seq and RNA-seq analysis demonstrated that *ASCL1*, *SOX2* and *NEUROD1* expression are shared master regulators between neuroendocrine cancers. *ASCL1* and *NEUROD1* expressions are mutually exclusive, and are drivers of intra-tumoral heterogeneity, a program that occurs due to distinct H3K27ac genome-wide de novo deposition in SCLC and PCa [192,193]. These data support the association of de novo cis-regulatory elements harboring H3K27ac as a determinant of lineage plasticity in neuroendocrine carcinomas. It is still unclear how EZH2 modulates gene transcription at different stages of PCa, and whether the processes described above interact with each other.

### 3.3. Cell Fate Determination

PcG proteins suppress alternate cell lineages when cells commit to differentiation by activating specific gene loci, and stem cells take on a new fate [194]. In this context, the role of epigenetics in the initiation and promotion of epithelial mesenchymal transition (EMT) has emerged. This idea was raised by the discovery of the role of EZH2 in promoting metastasis in cancer cells in “in vitro” experiments performed in human lung cancer cell lines [158]. These results were confirmed in melanoma mouse models, which showed a reduction in lymph node metastasis and the absence of lung metastasis in *EZH2*-conditional KO mice [195]. EMT is a conserved and reversible process, which promotes the transition and polarization of epithelial to mesenchymal cells, accompanied by an increase in the motility of the transformed cells. In epithelial cells, the EMT program is orchestrated by multiple signaling pathways, as well as epigenetic and post-translational modifications [196], and occurs in embryonic development, cancer, and wound healing. One of the principal characteristics of EMT cells is the loss of E-Cadherin, which mediates adherent junctions through homophilic bindings [197] and is accompanied by cell–cell adhesion disruption, together with an upregulation in Vimentin, fibronectin, and N-cadherin expression. Moreover, the expression of a wide variety of additional epithelial/mesenchymal markers, such as the SNAIL, ZEB, and TWIST families, is induced. Interestingly, EZH2, BMI1 and the histone demethylase (HDM) Lysine Demethylase 6B (KDM6B also called JMJD3), which can remove H3K27me3, have been associated with EMT, poor prognosis and metastasis in cancer [93,198,199,200]. EZH2 forms a complex with Snail and histone deacetylase (HDAC)1/HDAC2 to repress E-Cadherin expression and disable the homolog 2 interacting protein (DAB2IP) [199,201], while BMI1 is linked with Twist to also silence *E-Cadherin* expression as well as the tumor suppressor *p16INK4A* [198]. Moreover, it was shown that ZEB1 recruits SIRT1 to the *E-cadherin* promoter and deacetylates histone H3, which leads to reduced RNA polymerase II binding, leading to *E-cadherin* transcriptional loss [202]. In general, EZH2 promotes stemness and proliferation by silencing the expression of the transcription factors that determine cell fate.

## 4. Immune Regulation by EZH2 in TME

In addition to the direct impact of epigenetic changes on tumor cell growth and survival, it has been shown that epigenetic changes in tumoral cells mediate the immune responses of cells presented in the TME against the tumor. These mechanisms promote an immunosuppressive network, including the loss of expression of tumor-associated antigens or neoantigens, the impairment of cell surface antigen presentation, changes in the expression of immunosuppressive molecules and proinflammatory cytokines, and the aberrant expression of proteins associated with checkpoint pathways such as programmed cell death protein 1 (PD-L1) [203,204,205].

**Figure 4:**
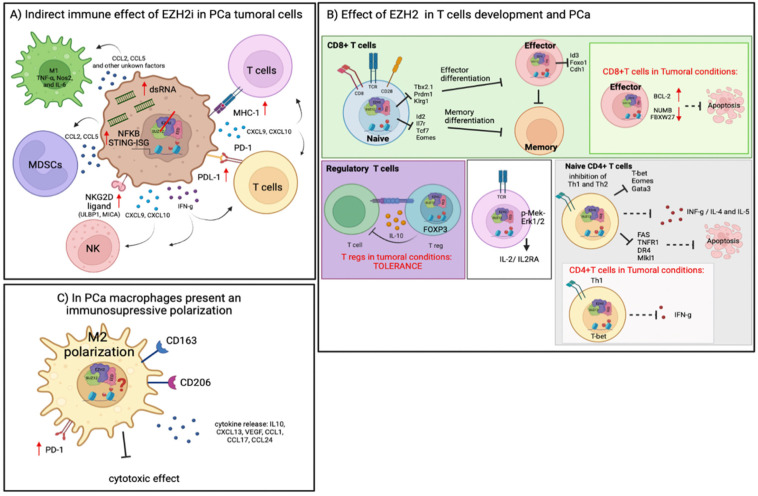
**Role of EZH2 in tumoral cells and tumor microenvironment (TME).** (**A**) The blockage of EZH2 in tumoral cells reduces the immunosuppressive EZH2-mediated mechanisms. Therefore, upon EZH2, the inhibition double-stranded (ds) RNA-STING-IFN stimulated genes (ISG) pathway is activated, promoting the increase in MHC class I (MHC-I) expression, an increase in antigen presentation, Th1 chemokine signaling and interferon response, the release of chemokines implicated in immune cells recruitment such as CXCL9 and CXCL10, as well as PDL-1 expression. However, the inhibition of EZH2 can lead to the accumulation of MDSCs in tumor sites. EZH2 is expressed in many immune cells, affecting cell function and differentiation. (**B**) In naïve CD8^+^ T-cells, EZH2-dependent gene repression silences both pro-effector and -memory genes to maintain developmental plasticity. In addition, in differentiated CD8^+^ effector T-cells, EZH2 selectively inhibits memory precursor signature genes, such as *Id3*, to restrict memory fate. In tumor-infiltrating CD8^+^ T-cells, EZH2 regulates BCL-2 expression, inhibiting apoptosis (green box). EZH2 expression in naive CD4^+^ T-cells represses *T-bet*, *EOMES* and *GATA3* expression, leading to the inhibition of Th1 and Th2 polarization (grey box). Moreover, EZH2 is required to stabilize *FOXP3* expression in regulatory T-cells (purple box). (**C**) In the PCa tumoral context, it has been reported that EZH2 expression by tumoral cells promotes M2 macrophage polarization, but how EZH2 regulates differentiation, polarization and migration in myeloid cells is unknown. Figure generated on the BioRender.com.

EZH2 is negatively associated with major histocompatibility complex (MHC) class I expression in different types of cancer [206]. In addition, it has been reported that EZH2 induces the silencing of secreted protein acidic and rich in cysteine (SPARCS), a specific type of IFN-γ inducing antisense 3′-UTR endogenous retroviral elements, resulting in double-stranded RNA generation and the engagement of IFN-γ antiviral signaling (MAVS) in mitochondria and the stimulator of interferon gene (STING) proteins [207]. Consistently, our previous studies indicated that EZH2 inhibition in PCa models activates a double-stranded RNA–STING–IFN stimulated genes (ISG) stress response that results in the upregulation of genes involved in antigen presentation, Th1 chemokine signaling and interferon response, including PD-L1, which is dependent on STING activation [208] (Figure 4A). In this context, a recent functional genomics screen in K562 leukemia cells [209] and studies in SCLC and TNBC [210,211] showed that EZH2 inhibition modulates the IFN-γ response, leading to MHC-I upregulation, and this effect is mediated by H3K27me3 repressive marks [206,210]. In addition, it has been shown that *MHC-I* genes are silenced by the recruitment of EZH2 by lincRNA EPIC1, inducing PD-1 treatment resistance [212]. These results indicate that EZH2 inhibition could be a therapeutically useful approach for the restauration of MHC-I expression. Moreover, in hepatocellular carcinoma and PCa, a negative correlation between *EZH2* and *PD-L1* was reported, which may result from the upregulation of H3K27me3 levels in the promoters of *CD274* and *IRF1* [208,213]. Consistent with this hypothesis, our previous studies showed that both the chemical and genetic inhibition of EZH2 in PCa organoids repressed H3K27m3 and induced the upregulation of *PD-L1* expression, indicating that EZH2 is involved in T-cell exhaustion mechanisms [208] (Figure 4A). Although the exact regulatory effect of EZH2 on immune CPIs therapy remains unclear, EZH2 inhibitors in combination with immunotherapy seem to be promising antitumor treatment strategies due to their synergistic effects in PCa mouse models [208], and in head and neck [214] and bladder cancer [215] studies. Moreover, in different cancer types, including melanoma [216] and ovarian [217], and in PCa mouse models [208], it has been shown that EZH2 inhibition leads to an increase in the intra-tumoral trafficking of activated CD8^+^ T-cells, and this recruitment is mediated by CXCL10-CXC chemokine receptor 3 (CXCR3) [203,218,219,220].

Further, the polarization of tumor-associated macrophages (TAMs), which play an important role in tumoral development, is influenced by cytokines and chemokines derived from tumoral cells (Figure 4A). Ongoing studies have demonstrated that EZH2 in tumoral cells affects TAMs via H3K27 methylation, but EZH2 inhibition has yielded contradictory results. For example, in hepatocellular carcinoma, miR-144/miR-451a is silenced by EZH2 and promotes M1 polarization, enhancing anti-tumor immunity [221]. In addition, EZH2 is inhibited in gliomas, mediated in a PRC2-dependent manner through miRNA-454-3p, immunosuppression or M2-like macrophage polarization [222]. However, macrophage infiltration is involved in the regulation of the LOX family protein (LOXL4) by EZH2-miR-29b/miR-30d in invasive breast cancer, and contributes to extracellular matrix remodeling [223]. Moreover, in SCLC, EZH2 mediates H3K27me3 in the enhancer region of *CCL2*, correlating with low macrophage infiltration [224], and promotes CCL5 production, which recruits M2 macrophages, facilitating metastasis and macrophage infiltration [225]. Moreover, our lab showed that EZH2 inhibition in PCa mouse models increased M1 TAMs (characterized by the expression of *TNF-α*, *Nos2*, and *IL-6*) with the concurrent loss of M2 TAMs (characterized by the expression of *Arg1* and *CD206*) [208,226] (Figure 4A). Together, these data indicate that the epigenetic changes promoted by EZH2 inhibition could affect TME, either indirectly (mediated by tumoral cells) or by direct modifications in TAMs. This is an effect that could lead to a tumor-specific response, promoting different tumoral microenvironments.

In addition to the immune dysfunction promoted by indirect epigenetic changes in tumoral cells, changes in the epigenetic landscape in immune cells can be presented in TME, including B (discussed above) and T-cells, macrophages, myeloid-derived suppressor cells (MDSCs), dendritic cells (DCs) and natural killer (NK) cells. Such chromatin remodeling within immune cells is demonstrated to promote the gradual loss of mature antigen-presenting functions and cytotoxic activity [227,228]. In the TME, signaling molecule pathways related with chronic inflammation, hypoxia and altered metabolism suffer functional epigenetic alterations, which influence the production of cytokines, chemokines, growth factors and adhesion molecules [229].

### 4.1. Role of EZH2 in T-Cell Differentiation and Cancer

Accumulating experimental evidence has shown that EZH2 mediates transcriptional regulation in T-cells, contributing to their development, divergent cell functions and proliferation, and may act as an immunomodulatory factor [141,144,230]. In naive CD4^+^ T-cells, the expression of transcriptional factors *T-bet* and *GATA3*, and Th1 and Th2 cytokines such as IFN-γ, IL-4 and IL-5, is repressed by EZH2, accompanied by the consequent inhibition of Th1 and Th2 polarization (review in [231]) (Figure 4B). In regulatory T-cells, EZH2 is required to stabilize *FOXP3* expression, and genetic or pharmacological EZH2 inhibition destabilizes its expression and promotes the immune-mediated rejection of tumors in mouse syngeneic models [232] (Figure 4B). Moreover, while in naive CD8^+^ T-cells, EZH2 silences both pro-effector and pro-memory genes to maintain developmental plasticity, in differentiated CD8^+^ effector T-cells, EZH2 selectively inhibits memory precursor signature genes [233] (Figure 4B). *EZH2*, *EED* and *SUZ12* are highly expressed in effector T-cells, suggesting a role in their differentiation process associated with an increase in H3K27me3 in promoter regions of memory-associated transcription factors TCF7 and EOMES [233]. In tumoral mouse models, tumor-infiltrating CD8^+^ T-cells expressed *EZH2,* and due to the upregulation of *BCL-2*, exhibited an anti-apoptotic feature associated with increased survival [234], which is abolished in *EZH2*-deficient CD8^+^ T-cells [235] (Figure 4B). Mechanistically, the suppressors *NUMB* and *FBXW27* are repressed by EZH2, leading to the activation of the Notch pathway, stimulating T-cell cytokine expression and survival via BCL-2 signaling [236]. In vitro experiments using B16F10 melanoma and human melanoma cells showed that in a co-culture with EZH2^−/−^ naive CD8^+^ T-cells or chemical EZH2 inhibition in human cells, T-cells maintained memory differentiation, and increased terminal effector differentiation compared with control samples, suggesting that effector T-cell populations may not be impacted by EZH2 inhibitors, at least in short treatment protocols [237]. Altogether, these findings highlight the importance of EZH2-mediated epigenetic regulation during CD8^+^ T-cell maturation; however, under certain circumstances EZH2 inhibitory therapy may have a negative impact on effector T-cells’ development, function, and survival. In addition, the loss of EZH2-mediated H3K27me3 in naive CD4^+^ T-cells at the *IFN-γ*, *EOMES* and *TBX21* loci resulted in the upregulation of IFN-γ production [227]. Therefore, the inhibition of EZH2 may transiently increase CD4^+^ T-cell effector function, but long treatments could impact the survival of effector CD4^+^ T-cells in TME. Thus, the regulatory role of EZH2 in T-cells should be taken into consideration in the development of combinatory therapies in cancer. Moreover, in tumoral conditions, in T-cell acute lymphoblastic leukemia (T-ALL), we have seen loss-of function mutations and deletions affecting EZH2 and SUZ12, leading to the hypomethylation of H3K27 target genes, including *Notch*, thereby contributing to oncogenesis. In addition, 25% of the T-ALL present global epigenetic remodeling towards H3K27ac, which activates mutations of the JAK/STAT signaling pathway [238]. On the contrary, the loss of SUZ12 in T-ALL disrupts the PRC2 complex, leading to H3K27me3 reductions, which promote open chromatin and the upregulation of the corresponding genes involved in oncogenic signaling pathways [239].

### 4.2. Role of EZH2 Function in NK Cells Differentiation and Cancer

NK cells share similar features of lytic granule exocytosis with cytotoxic T-cells in the elimination of cancer cells [240]. EZH2 plays a role in NK cell differentiation through the regulation of *HOXA9* and *HOXA10* [241]. Moreover, it has been shown that EZH2 inhibition leads to the repression of NKG2D ligands, including the gene expression of *ULBP1*, *MICA* and *MICB,* with the consequent activation of the cytotoxic effect of NK cells [242,243] (Figure 4A). Similarly, in muscle invasive bladder cancer models, EZH2 inhibition limits the proliferation of tumor cells in the context of KDM6A and SWI/SNF mutations [244]. Interestingly, EZH2 inhibitory treatment in tumoral models leads to the upregulation of genes associated with activated NK signaling, including *MIP-1α*, *ICAM1*, *ICAM2* and *CD86,* and the increased expression of IFN-γ.

### 4.3. Role of EZH2 Reprogramming Tumor Immunosuppressive Cells

It has been reported that epigenetic changes result in the abnormal differentiation and function of myeloid cells, promoting the development of a heterogeneous population of MDSCs with the capacity to suppress T-cell functions. Interestingly, the treatment of mouse syngeneic tumor models with EZH2 inhibitors resulted in the accumulation of CD11b^+^Gr1^+^ cells in the tumor tissue, an increase in M1 macrophages with the consequent decrease in M2 macrophages, as well as a reduction in IFN-γ-producing CD8^+^ and CD4^+^ T-cells [198] (Figure 4C). Interestingly, the depletion of MDSCs in TME promotes antitumor immune responses through the increased infiltration of functional T-cells [245]. These data highlight the divergent roles of EZH2 function in various immune cell subtypes, contributing to antitumor immune responses. However, the mechanistic understanding of how EZH2 regulates differentiation and migration in myeloid cells through DNA/histone methylation needs to be further elucidated.

## 5. Role of EZH2 in Tumoral Metabolism

Tumoral cells have a high level of metabolic requirements, different from those of normal cells, and upon tumoral growth the normal vasculature is not able to supply sufficient nutrients and oxygen. In the last decade, increasing evidence has suggested that epigenetic and metabolic alterations in cancer cells are interconnected and directly impact tumoral development and TME [124]. Under aerobic conditions, normal differentiated cells generate energy from glucose oxidative phosphorylation, but during tumor progression, even in aerobic environments, tumoral cells obtain oxygen mainly from glycolysis via the metabolization of glucose to lactic acid, a process denominated the “Warburg effect” [246]. In PCa, it has been reported that the overexpression of EZH2 regulates tumoral growth and the high rate of glycolysis in tumoral cells through miR-181b/hexokinase 2 (HK2) axis [247]. Moreover, Chip-seq analysis in glioblastoma cells has demonstrated that glycolysis and pyruvate-related genes, such as hexokinase 1 (*HK1*), *ENO2* and *PCK2,* are among the PRC-active target genes [248]. Supporting these results, in Pten Knockout PCa mouse models, the expression level of the glycolytic enzymes has been recently reported to be associated with the expression of luminal hypoxia-inducible factor 1 alpha (HIF-1α), and the expression of EZH2 and SOX2 in advanced stages of tumoral development. Moreover, luminal HIF-1α promotes the elevation of MDSCs-recruiting factors [249]. In addition, the metabolic reprograming in tumoral cells also involves lipid and amino acid metabolism [250,251], and in PCa, it has been shown that EZH2 is involved in the alteration of multiple metabolic pathways (reviewed in [250]). For example, *EZH2* depletion in PCa inhibited aerobic glycolysis, which was accompanied by the upregulation of miR-181b and lipoprotein-dependent lipid accumulation via the induction of ApoE expression in adipocytes [247]. In glioblastoma, it has been shown that EZH2 promotes lipid synthesis and the accumulation of fatty acid mediated thorough the activation of *PGC-1α* via the interaction between EZH2 and the mutant telomerase reverse transcriptase (TERT) [252]. Therefore, the metabolic properties of the TME, because of the cellular heterogeneity, result in specific subregions of metabolic stress, which include hypoxia, acidification, nutrient deficiency, and the accumulation of metabolic waste [250,253].

Like other cells, in immune cells, changes in the metabolism can lead to alterations in their functions and characteristics [254]. In the tumoral context, changes in metabolism promote alterations in the gene expression of macrophages and DCs through DNA methylation and acetylation [255]. Therefore, macrophages release different cytokines such as interleukins, including IL-6 and tumor necrosis factor-alpha (TNF-α), which plays a critical role during inflammation, damaged tissue repair, cell proliferation, invasion, and migration [256]. For example, the lack of glucose availability in tumoral conditions reduces the cytoplasmic NADH:NAD^+^ ratio, and promotes NAD(H)-sensitive transcriptional binding with the corepressor CtBP to p300, which blocks the binding of p300 and NF-κB with the proinflammatory gene promoters through the regulation of p65/RelA acetylation [257]. In glioblastoma, Wang M. et al. showed that the binding of Glial Cell-Derived Neurotrophic Factor (GDNF) to immune cell receptors (e.g., EGFR) promotes the activation of mTOR, contributing to the activation of the immune cell metabolism [258], and Yan et al. reported that the PI3K/AKT/mTOR pathway regulates HIF-1α, which is involved in reprogramming immune cell metabolism [259]. In addition, alterations in ornithine decarboxylase expression in macrophages lead to changes in the antimicrobial M1-like response by regulating histone modifications during inflammation [260]. Recently, in T-cells, it was reported that CD8^+^T-cells under tumoral conditions present disrupted methionine metabolisms and high expression levels of the transporter SLC43A2. Moreover, low intracellular levels of methionine and the methyl donor SAM in CD8^+^ T-cells were observed, and resulted in the loss of H3K79me2, which decreased *STAT5* expression levels and impaired T-cell immunity [261]. In addition, it has been shown that the lactate dehydrogenase A (LDHA) in activated T-cells supports aerobic glycolysis and promotes IFN-γ production [262]. In conclusion, the metabolic processes in tumor cells are affected by different and heterogeneous intracellular, epigenetic, and TME factors; therefore, tumors adapt to their environment, modulating their metabolic activities.

## 6. PRC2 Therapeutical Options in Cancer

Due to the key roles played by PRC2 in the pathophysiology of cancer development, controlling cellular plasticity in both tumoral and immune cells, several small molecules that modulate methyltransferase activity have been developed. The first pharmacological agents released directly targeted EZH2 activity. Since 2012, several potent and highly selective S-adenosyl-methionine-competitive inhibitors of EZH2 methyltransferase activity have been developed, and over 35 clinical trials have been initiated in different types of cancer (reviewed in [263]). However, researchers have also designed other molecules, including PRC2 activators, which target cancers governed by loss of function mutations, dual agents inhibiting EZH1/2, allosteric inhibitors binding to EED (which disrupt the EED–EZH2 protein–protein interaction), and compounds that induce the degradation of PRC2 constituent proteins.

### Clinical Trials Using PCR2 Therapy in Cancer

Different clinical trials have been undertaken to evaluate the efficacy of EZH2 inhibitors, dual agents, EED inhibitors and PRC2 degraders (Table 2 contains a summary of the clinical trials in progress) [264,265]. Despite the anti-tumor activity of EZH2 inhibitor that was previously demonstrated in refractory B-cell non-Hodgkin’s lymphoma and epithelioid sarcomas [266], the evaluation of GSK126, an EZH2 inhibitor, in a phase 1 clinical trial in patients with advanced hematologic and solid tumors showed insufficient evidence of clinical activity [267]. Using mouse models, it was demonstrated that the failure of this therapy could be associated with the presence of MDSCs in the TME because, when GSK126 was administered to immunocompetent hosts with depleted MDSC or in combination with MDSC-depletion drugs, including gemcitabine and 5-fluorouracil, the anticancer effect was potentiated [245]. As mentioned above, another mechanism of resistance for PRC2 inhibitors could be associated with mutations in the remodeling complexes SWI/SNF leading to an indirect reprograming of PRC2 in tumors [122,268]. The SWI/SNF complex is mutated in 20–25% of human cancers [119], and mechanistically, the loss of SNF5 tumor suppression, which has been identified in several cancers including chronic myeloid leukemia, malignant rhabdoid and central nervous system tumors, induces *EZH2* gene expression, and PRC2 target genes are broadly H3K27-trimethylated [122]. The first developed orally bioavailable EZH2 inhibitor was Tazemetostat. It was approved by the FDA in January 2020 for adults and pediatric patients with epithelioid sarcoma not eligible for complete resection, and patients with relapsed or refractory follicular lymphoma [264,269,270]. It is also currently being tested in clinical trials for diffuse large B-cell lymphoma, T-cell lymphoma, other non-Hodgkin’s lymphomas, and solid tumors [271]. In addition, TNBC and SCLC, which are highly mutated [272,273,274], and PCa, which is known as an immunologically “cold” tumor, rarely respond to immune checkpoint blockage (ICB) or PRC2 inhibitor monotherapy [267]. Moreover, a phase 2 clinical trial denominated Constellation’s, which studied the effects of the EZH2 inhibitor CPI-1205 in combination with enzalutamine or abiraterone/prednisone for mCRPC, was discontinued in 2020 due to a lack of efficacy [264]. These solid tumors often possess plasticity in both tumor- and TME-associated cells, and as mentioned above, MHC-I is transcriptionally suppressed by H3K27me3 modifications via PRC2 [206]. Thus, strategies to restore MHC-I expression could be a potential therapeutic approach in combination with other therapies. Due to their high heterogeneity, TNBC patients have been classified into four different subtypes, which present different global DNA methylation patterns. The mesenchymal subtype tumors have the lowest median of DNA methylation and expression levels of *MHC-I,* despite TNBC cells being only mildly sensitive to PRC2 inhibition. EZH2 and EED inhibition restored MHC-I expression and enhanced the efficacy of chemotherapy in mouse and in vitro models [210]. In addition, in SCLC, the epigenetic recovery of MHC-I is followed by a loss of neuroendocrine differentiation and the derepression of STING [211]. Therapeutically, EZH2 inhibition followed by STING agonism enhances T-cell recognition, and intratumoral T-cells increase in TNBC upon EZH2 inhibition in combination with taxane chemotherapy. In PCa, the repression of EZH2 results in the increased expression of interferon-stimulated genes (ISGs), which promote a favorable response to CPI. In this context, EZH2 inhibition activates a double-stranded RNA–STING–ISG stress response upregulating genes involved in antigen presentation, Th1 chemokine signaling, interferon response, the intratumoral trafficking of activated CD8^+^ T-cells, and increased M1 tumor-associated macrophages [208]. These studies identify EZH2 as a potent inhibitor of antitumor immunity and responsiveness to CPI. These preclinical studies support the results obtained in the clinical trial combining CPI-1205 with the anti-CTLA-4 antibody (ipilimumab), which was started because Goswami et al. showed that EZH2 inhibition modifies the function of regulatory T-cells, promoting an effector-like T-cell response, which leads to increased antitumor immunity [275]. This study showed that anti-CTLA-4 therapies lead to the upregulation of EZH2; therefore, the combination of EZH2 inhibition with ipilimumab enhances efficacy in tumor-bearing mice [275], providing a rationale for combining both therapies. Moreover, protein degradation therapies have recently become a promising therapeutical option in cancer because these strategies target the non-histone methylation activity functions of EZH2 discussed above. Currently there are two molecules under development [276,277,278] for the treatment of PCa and breast cancer. The first targeted degrader of EZH2 showed promising results as it was demonstrated to be efficient in the degradation of EZH2 in different cancer cell lines, including the non-malignant PCa cell line PNT2 [276].

## 7. Conclusions

In this review, we have summarized emerging evidence showing that EZH2 is expressed in cancer, and most of the immune cells forming the TME. These discoveries suggest that immunotherapy resistance could be associated with the coordinated silencing of genes involved in antigen processing, macrophage polarization, T-cell reprograming and cytokine secretion, which can be reversed by EZH2 inhibitors. Therefore, the combination of drugs targeting the pro-oncogenic role of EZH2 in tumoral cells and its role promoting an immunosuppressive TME, with immunotherapy, could have a synergistic effect, improving the therapeutic responses of some types of cancer. Despite preclinical studies in hepatocellular carcinoma [279], PCa [208], head and neck cancer [280] and Ewing sarcoma [281] showing that the combination of EZH2 inhibitors and CPIs has a synergistic effect, there are no studies investigating the immunological consequences of these combinations. EZH2 activity is fundamental to maintaining body homeostasis and to the development of different cells, including lymphoid cells, performing critical immune effector functions against tumors. For these reasons, the efficacy and safety of the different possible combinations should be carefully analyzed in the context of each specific cancer type, tumor stage and correct dosage, so as to avoid possible toxic effects. Given the complex and dynamic relationship between tumoral cells and the TME, it will be necessary to develop tools to identify patients that will derive more benefits from the combinatory therapy, as well as tools to follow-up correctly the therapeutic response.

## Figures and Tables

**Table 1:** PRC1 and PRC2 components in different species.

	Mammals	Drosophila	Arabidopsis	Characteristic Domain	Activity
PRC1	RING1A/RING1B Bim-1	dRing/Sce	AtRING1A/AtRING1B	RING finger domain [49]	E3 ubiquitin ligase activity for H2A
	PCGF1-6	Psc	AtBMI1A/ AtBMI1B/ AtBMI1C	RING finger domain	Co-factors for H2A monoubiquitination
	CBX2/4/6/7/8	Pc	EMF1 LHP1(TFL2)VRN1 VAL1/2/3	Chromodomain	Recognizes and binds to H3K27me3
	PHC1/ PHC2/ PCH3	Ph	UNKNOWN	Sterile Alpha Motif (SAM) domain and Zinc finger domain	Mediates monoubiquitinaiton of histone H2A [50]
PRC2	EZH1/2	E(z)	CLF/ SWN/ MEA	SET domain	H3K27 methyltransferase [51]
	SUZ12	Su(z)12	EMF2/ VRN2/ FIS2	Zinc finger	Mediates core PRC2 and accessory components’ interaction [51]
	EED	Esc	FIE	WD-40 repeat domain	Stabilizes and enhances E(z) [52]
	RBAP48/46	P55/Nurf55	MSI1-5	WD-40 repeat domain	Binds to histones and Su(z)12
	EZHIP			tissue-specific cofactor of PRC2	Limits PRC2-mediated H3K27me3 deposition [53]
PRC2.1	PCL1/2/3	Pcl		PHD finger, TUDOR	Promotes PRC2 recruitment to CpG islands that lack H3K27m3 mark
	EPOP or PALI1/2				
PRC2.2	JARID2			Zinc finger, ARID domain, JmjC and JmjN	Promotes the PRC2 recruitment to chromatin that has PcG-dependent modifications
	AEBP2			Zinc finger	

**Table 2:** Summary of the clinical trials using PCR2 therapy in cancer.

Subgroup	Compound	Clinical Trial	Phase	Clinical Trial Identifier	Status
EZH2 inhibitors	Tazemetostat	Patients with relapsed, refractory follicular lymphoma	III	NCT04224493	Recruiting
		In combination with pembrolizumab for patients with locally advanced or metastatic urothelial carcinoma	I/II	NCT03854474	Recruiting
		Patients with moderate and severe hepatic impairment with advanced malignancies	I	NCT04241835	Recruiting
		Patients with refractory B-cell non-Hodgkin’s lymphoma with EZH2 gene mutation	II	NCT03456726	Active
		Patients with recurrent ovarian or endometrial cancer	II	NCT03348631	Suspended
		Patients with B-cell lymphoma or advance solid tumors	I	NCT03010982	Completed
		Patients with mCRPC (+abiraterone/prednisone or enzalutamide)	II	NCT02875548	Ongoing
		Patients with advanced epithelioid sarcoma in combination with doxorubicin	III	NCT04204841	Recruiting
		Prelapsed or refractory INI-1 negative tumors or synovial sarcoma, rhabdoid tumors, malignant rhabdoid tumors of ovary	I	NCT02601937	Recruiting
		In combination with Atezolizumab and Obinutuzumab in relapsed/refractory follicular Lymphoma and diffuse Large B-cell Lymphoma	I	NTC02220842	Completed
		In combination with doxorubicin and HCI for advanced soft-tissue sarcoma or epitheloid sarcoma	III	NCT04204941	Recruiting
	CPI-1205	ProSTAT: Patients with mCRPC in combination with abiraterone/prednisone (ARPI)	II	NCT03480646	Recruiting
		ORIO-E: Patients with advanced solid tumors in combination with ipilimumab	I/II	NCT03525795	Recruiting
		Patients with B-cell lymphomas	I	NCT02395601	Completed
		Hepatic impairment advanced malignant solid tumor	I	NCT04241835	Recruiting
	CPI-0209	Patients with advanced solid tumors in combination with irinotecam	I/II	NCT04104776	Recruiting
	Valemetostat	Patients with acute myelogenous leukemia or acute lymphocytic leukaemia	I/II	NCT03110354	Completed
		Patients with recurrent SCLC in combination with irinotecam	I/II	NCT03879798	Recruiting
		Patients with non-Hodgkin lymphoma (NHL)	I	NCT02732275	Active
		Patients with relapsed/refractory adult T-cell leukaemia or lymphoma	II	NCT04102150	Active
		Participants with hepatic impairments (single-dose)	I	NCT04276662	Completed
	PF-06821497	Patients with follicular lymphoma, CRPC and relapsed/refractory small cell lung cancer (SCLC)	I	NCT03460977	Recruiting
	SHR2554	Relapsed or refractory mature lymphoid neoplams	I	NCT03741712	Recruiting
	SHR2554	In combination with SHR3680 (inhibits androgen-mediated translocation of AR) in CRPC	I/II	NCT03460977	Recruiting
	EPZ-6438+ARPI	CELLO-1: Patients with mCRPC who have not received chemotherapy	I/II	NCT04179864	Recruiting
Dual EZH2 inhibitors	DS-3201b	Patients with hepatic impairment (single dose DS-3201b)	I	NCT04276662	Recruiting
		Patients with adult T-cell leukemia/lymphoma	II	NCT04102150	Recruiting
		Patients with SCLC with irinotecam	I/II	NCT3879798	Recruiting
		Patients with lymphomas	I	NCT02732275	Recruiting
EED inhibitors	MAK683	Patients with advanced malignancies (diffuse large B cell lymphoma)	I/II	NCT02900561	Recruiting

## Data Availability

Not applicable.
